# The CaSR Modulator NPS-2143 Reduced UV-Induced DNA Damage in Skh:hr1 Hairless Mice but Minimally Inhibited Skin Tumours

**DOI:** 10.3390/ijms24054921

**Published:** 2023-03-03

**Authors:** Chen Yang, Mark Stephen Rybchyn, Warusavithana Gunawardena Manori De Silva, Jim Matthews, Katie Marie Dixon, Andrew J. A. Holland, Arthur David Conigrave, Rebecca Sara Mason

**Affiliations:** 1 School of Medical Sciences and Bosch Institute, University of Sydney, Sydney, NSW 2006, Australia; 2 School of Chemical Engineering, University of New South Wales, Sydney, NSW 2033, Australia; 3 Sydney Informatics Hub, University of Sydney, Sydney, NSW 2008, Australia; 4 Douglas Cohen Department of Paediatric Surgery, The Children’s Hospital at Westmead Clinical School, The Faculty of Medicine and Health, The University of Sydney, Sydney, NSW 2145, Australia; 5 School of Life and Environmental Sciences, Charles Perkins Centre (D17), University of Sydney, Sydney, NSW 2006, Australia

**Keywords:** ultraviolet radiation (UV), photoprotection, 1a,25-dihydroxyvitamin D3 (1,25D), cyclobutane pyrimidine dimer (CPD), 8-hydroxy-2′–deoxyguanosine (8-OHdG), calcium sensing receptor (CaSR), photocarcinogenesis, cyclic AMP response element binding factor (CREB), squamous cell carcinoma (SCC)

## Abstract

The calcium-sensing receptor (CaSR) is an important regulator of epidermal function. We previously reported that knockdown of the CaSR or treatment with its negative allosteric modulator, NPS-2143, significantly reduced UV-induced DNA damage, a key factor in skin cancer development. We subsequently wanted to test whether topical NPS-2143 could also reduce UV-DNA damage, immune suppression, or skin tumour development in mice. In this study, topical application of NPS-2143 (228 or 2280 pmol/cm^2^) to Skh:hr1 female mice reduced UV-induced cyclobutane pyrimidine dimers (CPD) (*p* < 0.05) and oxidative DNA damage (8-OHdG) (*p* < 0.05) to a similar extent as the known photoprotective agent 1,25(OH)_2_ vitamin D3 (calcitriol, 1,25D). Topical NPS-2143 failed to rescue UV-induced immunosuppression in a contact hypersensitivity study. In a chronic UV photocarcinogenesis protocol, topical NPS-2143 reduced squamous cell carcinomas for only up to 24 weeks (*p* < 0.02) but had no other effect on skin tumour development. In human keratinocytes, 1,25D, which protected mice from UV-induced skin tumours, significantly reduced UV-upregulated p-CREB expression (*p* < 0.01), a potential early anti-tumour marker, while NPS-2143 had no effect. This result, together with the failure to reduce UV-induced immunosuppression, may explain why the reduction in UV-DNA damage in mice with NPS-2143 was not sufficient to inhibit skin tumour formation.

## 1. Introduction

Skin cancers can be categorized into three main types: (i) basal cell carcinoma (BCC); (ii) squamous cell carcinoma (SCC), both of which arise from keratinocytes; and (iii) melanoma. Pathological changes in skin, including ultraviolet radiation (UV)-induced DNA damage [1,2,3], mutagenesis [4], inflammation [5], and immunosuppression [6,7] can ultimately lead to photocarcinogenesis. UV not only directly induces DNA lesions such as cyclobutane pyrimidine dimers (CPDs) and (6–4) photoproducts [8], but also induces indirect biological damage targeting DNA, protein, and lipids via the production of reactive oxygen species (ROS) and nitric oxide products, forming 8-hydroxy-2′-deoxyguanosine (8-OHdG) as a marker of oxidative DNA damage [9]. UV has potent immunosuppressive effects that promote tumour development [7,10,11,12].

Repetitive UV-induced epidermal thickening and pigmentation production together protected mice [13] and humans from subsequent UV challenges, with 75% less erythema and 60% less DNA damage in skin [14,15]. This thickening of the skin as a result of keratinocyte differentiation and may be more protective than melanogenesis (pigmentation production) in response to UV, at least in some populations [16]. Calcium concentration is believed to act as a switcher between proliferation and differentiation of keratinocytes [17]. This is consistent with a well-defined gradient for total calcium that increases from the basal to the outermost layers of the epidermis [18]. The responses of keratinocytes to extracellular calcium ion concentrations (Ca^2+^_o_) and the maintenance of systemic calcium homeostasis are mainly controlled by the calcium-sensing receptor (CaSR), a member of family C of the G protein-coupled receptors (GPCR) [19,20]. There are commercially available small molecule allosteric agents, for example, NPS-2143 works as an antagonist that reduces CaSR activity to block the increase of Ca^2+^_i_ [21,22,23,24,25]. NPS-2143 has been used in an attempt to promote a brief secretion of parathyroid hormone in plasma for treatment of osteoporosis [22]. Previously we reported that CaSR knockdown or exposure to the CaSR negative allosteric modulator NPS-2143 protected human keratinocytes in culture against UV-induced DNA damage at a similar level to the known photoprotective agent, 1,25-dihydroxyvitamin D_3_ (1,25D) [26]. This photoprotective activity of NPS-2143 was attributed at least in part to enhanced DNA repair and to reduction in ROS [26].

Immunosuppression, along with UV-induced DNA lesions, is a key factor leading to photocarcinogenesis [27]. Topical 1,25D has been shown to protect mice from UV-induced CPDs, apoptotic sunburn cells, and UV-induced immunosuppression, and to reduce UV-induced skin tumours [28,29,30,31] as well as chemically-induced skin tumours [32]. The use of albino hairless (Skh:hr1) mice exposed to chronic UV is accepted as a reliable model of photocarcinogenesis [33,34,35]. While cultured primary keratinocytes provide a powerful approach for studying epidermal biology, they imperfectly model the multi-cell types and structural order of living epidermis [36]. Thus we aimed to investigate, for the first time in a mouse model, whether manipulation of the CaSR by its negative allosteric modulator NPS-2143 would protect against DNA damage in mouse epidermis after acute UV exposure. We also wanted to examine if topical treatment of NPS-2143 would reduce UV-induced skin inflammation and immune suppression, as well as in response to a chronic UV-exposure, whether it would reduce UV-induced skin tumours in comparison with the positive control, 1,25D.

## 2. Results

### 2.1. NPS-2143 Protects against DNA Damage and Apoptotic Keratinocytes in Skh:hr1 Mice

**Figure 1:**
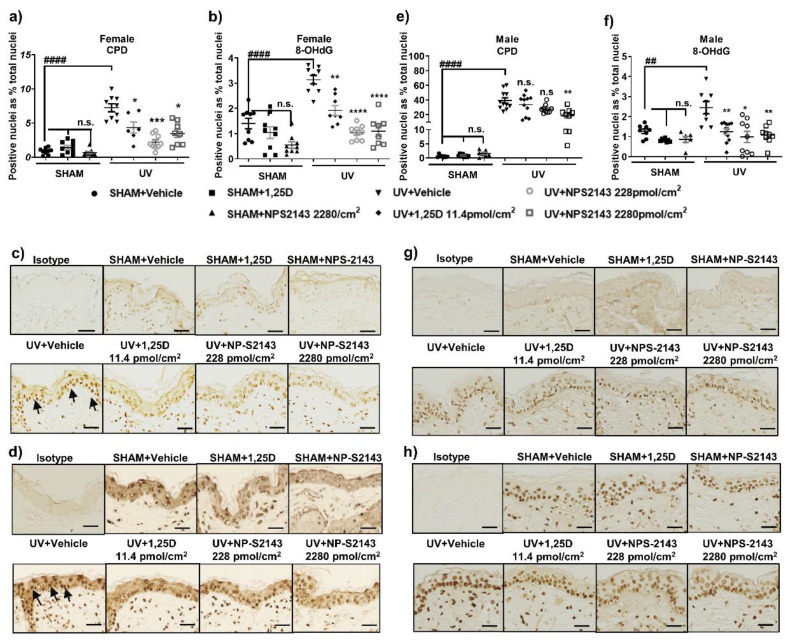
NPS-2143 inhibits DNA damage in female and male mouse skin following acute UV irradiation. Immediately after exposure to ssUV, female and male Skh:hr1 mice were treated with vehicle 1,25D (11.4 pmol/cm^2^) or NPS-2143 (228 or 2280 pmol/cm^2^) topically and the skin was harvested after 3 h. DNA damage is shown in graphs for CPD in female (**a**) or male (**e**) mice and 8-OHdG in female (**b**) or male (**f**) mice with graphs displaying data as dot plots. These graphs show representative data from a minimum of two individual experiments with similar results on each occasion. **** *p* < 0.0001, *** *p* < 0.001, ** *p* < 0.01, * *p* < 0.05, and n.s. not significant, when compared with UV-vehicle treated group by linear mixed model analysis; ^##^ *p* < 0.01, ^####^ *p* < 0.0001 significantly different from sham vehicle by *t*-test (**a**): *t* = 6.088, df = 10 (**b**): *t* = 5.385, df = 10 (**c**): *t* = 6.084, df = 13, (**d**): *t* = 2.728, df = 10. *n* = 9 (triplicate biopsies per mice, 3 mice per group). Photomicrographs show UV-induced CPD in female (**c**) or male (**g**) mice and 8-OHdG in female (**d**) or male (**h**) mice. Black arrows point to the dark brown staining in nuclei indicating the presence of DNA damage. Scale bar = 100 μm.

**Figure 2:**
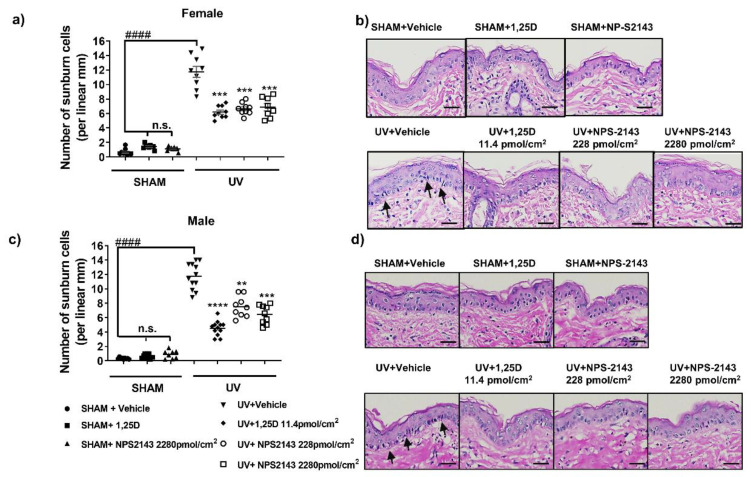
NPS-2143 reduces apoptotic keratinocytes in female and male mouse skin following acute UV irradiation. Immediately after exposure to ssUV, female and male Skh:hr1 mice were treated with vehicle 1,25D (11.4 pmol/cm^2^) or NPS-2143 (228 or 2280 pmol/cm^2^) topically, and skin was harvested after 3 h. The number of sunburn cells in female (**a**) and male (**c**) mice is presented as displaying data as dot plots. These graphs show representative data from two individual experiments with similar results on each occasion. **** *p* < 0.0001, *** *p* < 0.001, ** *p* < 0.01, and n.s. not significant compared with UV-vehicle treated group by linear mixed model analysis; ^####^ *p* < 0.0001 significantly different from UV-vehicle by *t*-test (**a**): *t* = 8.417, df = 10 (**c**): *t* = *t* = 12.09, df = 14. *n* = 9 (triplicate biopsies per mice, 3 mice per group). (**b**,**d**) Photomicrographs of UV-induced sunburn cells in female (**b**) or male (**d**) mice. Arrows show histological appearance of apoptotic sunburn cells in haematoxylin and eosin stained Skh:hr1 mouse skin section with shrunken, elongated nuclei-stained dark purple. Scale bars =100 μm.

Acute UV irradiation generated CPDs, oxidative DNA damage 8-OHdG (Figure 1), and sunburn cells (Figure 2) in mouse skin. The photoprotective hormonal form of vitamin D, 1,25D [29,37,38,39,40,41], was used as the positive control in these experiments. All agents in all experiments were applied topically immediately after exposure to solar-simulated UV (ssUV). Minimal staining in SHAM skin, particularly of 8-OHdG, indicates basal damage of the nuclei. In female mice, topical NPS-2143 at 2 concentrations, 228 pmol/cm^2^ and 2280 pmol/cm^2^ effectively reduced both CPD (*p* < 0.05, F(2.062, 19.25) = 15.64) and 8-OHdG (*p* < 0.05, F(1.537, 11.78) = 34.58) (Figure 1a–d). Topical NPS-2143 also reduced UV-induced sunburn cells (*p* < 0.01, F(2.044, 15.67)= 32.04) which are apoptotic keratinocytes with characteristic pyknotic nuclei and eosinophilic cytoplasm [42] (Figure 2a,b).

In male Skh:hr1 mice, significant reduction in UV-induced CPD was only seen after treatment with a high dose of NPS-2143, 2280 pmol/cm^2^ (*p* < 0.01, F(2.190, 18.98) = 6.738) (Figure 1e–h). Both concentrations of this agent, however, as well as 1,25D, significantly protected against oxidative DNA damage, 8-OHdG (*p* < 0.01, F(1.284, 14.55) = 8.726), in males (Figure 1f,h), and against sunburn cells (*p* < 0.01, F(2.663, 33.74) = 53.07) (Figure 2c,d).

### 2.2. NPS-2143 Effects on Inflammatory Response after ssUV and on Contact Hypersensitivity in Female Mice

**Figure 3:**
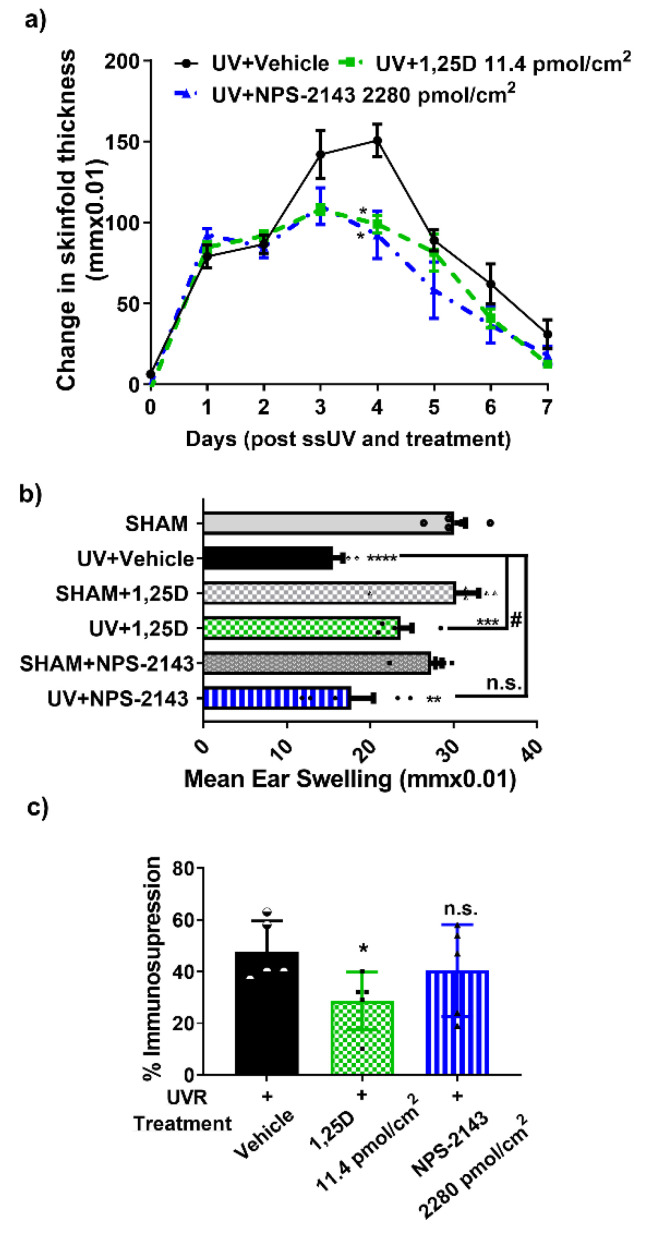
The effect of NPS-2143 on ssUV-induced inflammatory edema and the contact hypersensitivity reaction to oxazolone in female Skh:hr1 mice. (**a**) Measurement of edema daily (days 1—7) by mean change in dorsal skinfold thickness ± SEM compared to non-irradiated skinfold thickness. * *p* < 0.05 when compared with UV Vehicle treated group. *n* = 5 mice per group by linear mixed model analysis. (**b**) Ear Swelling, **** *p* < 0.0001 *** *p* < 0.001, ** *p* < 0.01 significantly different from SHAM group, ^#^ *p* < 0.05, n.s. not significant compared with UV-vehicle-treated group, by linear mixed model analysis, *n* = 5 mice per group. (**c**) SSUV-induced immunosuppression, * *p* < 0.05, n.s. not significant compared with UV-vehicle-treated group by linear mixed model analysis, *n* = 5 mice per group.

After exposure to 3 minimal erythemal doses (MED) of solar-simulated UV, where a MED is defined as the lowest dose of UV which produces a mild reddening of the skin at 24 h, the mice developed skin edema [31]. In this study, skinfold thickness increased daily, reaching a maximum at day 4 post-UV, then decreased gradually (Figure 3a). On the 4th day, UV-induced edema was significantly reduced in the presence of 1,25D (11.4 pmol/cm^2^) (*p* < 0.05) or NPS-2143 (2280 pmol/cm^2^) (*p* < 0.05), compared to the vehicle-treated control mice (F(1.531,9.187) = 8.857).

In order to study how UV exposure affects contact hypersensitivity to oxazalone, female mice were exposed to ssUV or SHAM, then treated topically with the various agents. One week later, all the mice were sensitized with 2% oxazolone applied to the non-irradiated abdominal skin. The mice were then challenged one week after this, by topical application of oxazalone to the ears to trigger swelling. Ear thickness measurements were taken before the challenge and again 18 h later. The average ear swelling expressed as the difference between ear thickness measured before and after challenge (at 18 h) in the non-UV exposed (SHAM) vehicle-treated mice was 293 ± 45 microns, and there were no differences among all non-irradiated groups (Figure 3b). In the UV-irradiated vehicle-treated mice, the average ear swelling of vehicle-treated mice was 155 ± 30 microns, indicating significant suppression of the immune response. With topical treatment with 1,25D, average ear swelling after UV was 217 ± 52 microns (Figure 3b), consistent with partial restoration of the contact hypersensitivity response. Though this swelling in response to oxazolone was smaller than in the SHAM with 1,25D-treated mice, the response was significantly better than in the vehicle-treated UV-exposed mice (*p* < 0.05, F (3.172, 28.55) = 21.73). Mice treated with NPS-2143 and UV had a measured average ear swelling of 177 ± 63 microns, not significantly different from vehicle-treated, UV exposed mice (Figure 3b). When calculated as a percent immune suppression after UV [31], the values were 52% immune suppression in the vehicle-treated group, 26% in the 1,25D-treated group (*p* < 0.05 vs vehicle-treated), and 39% in the mice treated with NPS-2143 (n.s. vs vehicle-treated mice) (F (1.582, 21.36) = 3.405) (Figure 3c).

### 2.3. Study of NPS-2143 in Photocarcinogenesis in Female SKh:hr1 Mice

**Figure 4:**
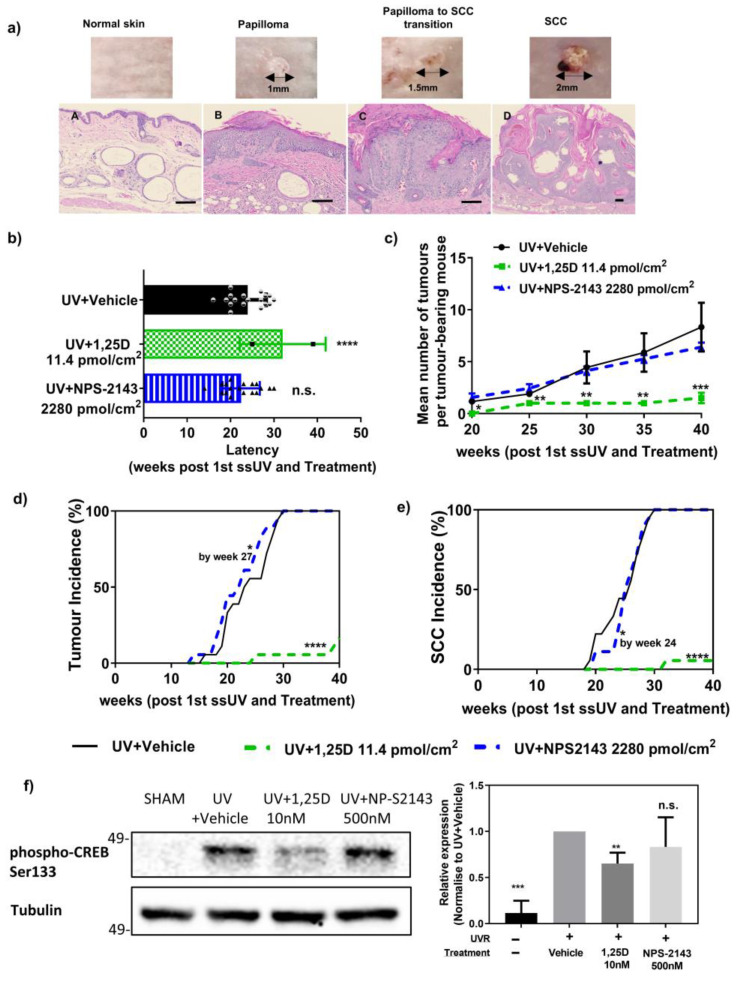
Effect of NPS-2143 in chronic SSUV-induced photocarcinogenesis in Skh:hr1 mice. (**a**) Photographic and photomicrographic examples of (**A**) normal skin, (**B**) papilloma, (**C**) papilloma to SCC transition, and (**D**) SCC as seen in this study. Scale bar = 100 μm. (**b**) Tumour Latency (**c**) Tumour Multiplicity **** *p* < 0.0001, *** *p* < 0.001, ** *p* < 0.01, n.s. not significantly different from UV +Vehicle group. (**d**) Tumour Incidence (**e**) SCC incidence * *p*< 0.05, **** *p* < 0.0001 significantly different from UV + Vehicle group at indicated week by Mantel–Cox test. Each of these graphs illustrates data from a single experiment (*n* = 18) and is presented as means ± SEM. (**f**) Western Blot and densitometry of p-CREB and tubulin (loading control). Human keratinocytes cultured in 96 well plates were irradiated with 400 mJ/cm^2^ UVB followed by treatment with vehicle, 10 nM 1,25(OH)_2_D_3_ or 500 nM NPS-2143 in the presence of 1 mM CaCl_2_ for 90 min. Densitometry of triplicate blots (Figure S1a) for p-CREB expression (Mean + SD) was normalized to UV + Vehicle and shown as a relative expression. Statistical significance was calculated with GraphPad prism using a one-way ANOVA followed by Tukey’s test. **p < 0.01, *** *p* < 0.001, n.s. not significant when compared with vehicle-treated cells after UV.

Albino hairless Skh:hr1 mice develop papillomas and then SCC after 10 weeks of chronic ssUV exposure [31,34,43]. During the 40 weeks of study, tumours normally appeared as small papillomas which gradually increased in diameter (Figure 4a). Papillomas are a benign outgrowth of skin in mice, comparable to the onset of actinic keratoses (AK) in humans [44]. A proportion of these papillomas showed signs of progression towards malignancy. These may be identified grossly and verified histologically as squamous cell carcinomas in later weeks (Figure 4a) [34].

- ***Tumour latency***

The onset of detectable tumour (papilloma) formation in mice varied between treatment groups. As shown in Figure 4b, the latency in the vehicle-treated group was 24.0 ± 1.0 weeks. A significantly increased latency of 33.6 ± 2.5 weeks (*p* < 0.0001, F(2, 35) = 2.560) was seen in 1,25D-treated mice (11.4 pmol/cm^2^). The average latency for NPS-2143-treated mice (2280 pmol/cm^2^) was 22.4 ± 1.0 weeks, which was not significantly different from the vehicle control (Figure 4b).

- ***Tumour multiplicity***

Tumour multiplicity including both papillomas and SCCs was calculated at each weekly time point, as the average number of tumours per tumour-bearing mouse. Figure 4c shows tumour multiplicity throughout the 40-week study. Vehicle- and NPS-2143 (2280 pmol/cm^2^)-treated mice showed a steady increase in tumour multiplicity from week 16 to week 40, while 1,25D-treated mice (11.4 pmol/cm^2^) had remarkably lower tumour multiplicity. Compared to vehicle-treated mice, tumour multiplicity was significantly reduced in the 1,25D-treated group at all week-points assessed (*p* < 0.05 at week 20, *p* < 0.01 at week 25, 30 and 35, *p* < 0.005 at week 40, F(2, 45) = 2.255). However, there was no significant difference between NPS-2143 treated and vehicle-treated mice.

- ***Tumour incidence***

Progressive total tumour incidence including both papillomas and SCCs was calculated each week as the percentage of mice in each group bearing at least one tumour, as shown in Figure 4d. The incidence data were analysed statistically using a Mantel–Haenszel log-rank test (Mantel–Cox test) [45], in which all treatments were compared to vehicle-treated mice at 27 weeks and after (Table 1a). This analysis reveals whether there was a difference in the risk of developing a tumour. Mice treated with 1,25D (11.4 pmol/cm^2^) had significantly reduced tumour incidence compared with the vehicle-treated group throughout the entire experiment (Figure 4d green dotted line, Table 1a, Mantel–Cox test Chi-square Value = 27.09, df = 1). NPS-2143-treated (2280 pmol/cm^2^) mice demonstrated a time point-dependent increase in total tumour incidence compared to the vehicle-treated group at 27 weeks after the first irradiation, but by 28 weeks and over the subsequent period until 40 weeks, there was no significant difference (Figure 4d blue dotted line, Table 1a, Mantel–Cox test Chi-square Value = 4.061, df = 1).

Mice developed squamous cell carcinomas (SCCs) throughout the study from 18 weeks. The SCC-only incidence is shown in Figure 4e. Mice treated with 1,25D (11.4 pmol/cm^2^) had significantly reduced SCC incidence compared with the vehicle control group throughout the experiment (Figure 4e green dotted line, Table 1b, Mantel–Cox test Chi-square Value = 5.355, df = 1). Only one mouse in the group of 18 (5.5%) treated with 1,25D developed an SCC at week 32 and this was still present at the end of the study. NPS-2143-treated mice had a significantly lower risk of developing SCC (5 out of 18, 27.8%) compared to the vehicle-treated group (8 out of 18, 44.4%) at the 24th week post-irradiation (Figure 4e Blue dotted line, Table 1b, Mantel-Cox test Chi-square Value = 22.09,df = 1). However, from the 25th week until the end of the experiment, there was no significant difference between the risk of SCC in NPS-2143- and vehicle-treated mice (Table 1b).

- Phosphorylation of cyclic AMP response element binding protein (CREB) as a predictor of anti-tumour activity

After UV exposure, CREB phosphorylation in epidermal cells increases and this has been proposed as a marker of tumour promoting activity [46]. In this study, 1,25D significantly reduced the risk of developing papillomas and SCC compared with the vehicle-treated group, while NPS-2143 had no overall effect on tumour or SCC incidence (Figure 4e, Table 1b).

Phosphorylation of CREB after UV, 1,25D, or NPS-2143 was studied in normal human keratinocytes. Negligible basal phospho-CREB (p-CREB) was seen in non-irradiated keratinocytes (SHAM) (Figure 4f). In cultured human keratinocytes, exposure to ssUV increased p-CREB measured 90 min after exposure (Figure 4f). Treatment of the cells immediately after UV with 1,25D significantly reduced p-CREB while treatment with NPS-2143 had no effect whether expressed as a function of tubulin as loading control (Figure 4f, F (3, 8) = 0.8378, and Figure S1a,) or as a function of total CREB (Figure S1b).

A summary of the main differences between responses to the positive control 1,25D and NPS-2143 is shown below (Table 2).

## 3. Discussion

In this study, the CaSR negative allosteric modifier NPS-2143, like the positive control 1,25D, when applied topically immediately after ssUV, effectively reduced UV-induced DNA lesions of CPD and 8-OHdG in female Skh:hr1 mice. Both NPS-2143 and 1,25D reduced oxidative DNA damage in male mice and at the higher concentration, NPS-2143 also reduced CPD in male mice. These results are consistent with the findings from our study using keratinocytes in primary culture from male human donors [26]. This is a discovery of a photo-protective role for NPS-2143, entirely different from its better recognised role as a therapeutic agent for raising parathyroid hormone levels. NPS-2143 negatively modulates the affinity of the CaSR for extracellular Ca^2+^, thereby reducing its activity [21,22,23,24,25]. In order to better discriminate the role of the CaSR in this study, it would have been useful to examine a CaSR antagonist (NPS2390 or Calcium-Sensing Receptor Antagonists I), but these studies were beyond the resources available for this work.

Sunburn cells and apoptotic keratinocytes were observed as soon as 3 h after acute exposure to UVB [47], despite being cells that undergo programmed cell death as a result of extensive and irreparable DNA damage [42]. It is reasonable to propose that reduced DNA damage, along with increased DNA repair [21] in the presence of NPS-2143, led to fewer apoptotic keratinocytes in mouse skin. In the meantime, we also observed improved survival of human keratinocytes in culture after UV exposure in the presence of NPS-2143 (Figure S2). It is likely that reduced generation of ROS, as previously reported with NPS-2143 after UV [21], contributed to reduced apoptosis. While sunburn cells are an index of apoptosis, analysis of more specific markers of apoptosis such as caspase3/7 or cleaved PARP in the mouse tissue would indicate early stages of apoptotic events and could help to elucidate the mechanism.

The reductions in 8-OHdG in male mice with 1,25D or NPS-2143 were similar to those seen in female mice. However, only the higher dose of NPS-2143 reduced CPD in male mice. Resistance in male mice to protection against UV-induced CPD in the presence of 1,25D has been previously reported in a separate study [47]. In that study, we demonstrated that the estrogen receptor-β (ER-β), the only estrogen receptor present in female mouse skin, seemed likely to be involved in reductions in CPD with 1,25D, since treatment with an ER-β antagonist or the use of female ER-β knockout mice reduced the response to 1,25D [47]. The current results indicate less effective protection against UV-induced CPD by a negative allosteric modulator of the CaSR. Whether this is also related to the presence of ER-β in female mouse skin or some other sex-related difference is an interesting question but was beyond the scope of the current study. A simple explanation for the reduced effectiveness of the lower dose of NPS-2143 could be that male mice have approximately 20% thicker skin than female mice, regardless of UV [48]. DNA damage is a major contributor to UV-induced immune suppression [49] and susceptibility of male mice or humans to UV-induced immune suppression is greater than in their female counterparts [50,51]. Male mice are more susceptible than females to photocarcinogenesis [52], whereas incidence and mortality of skin cancers is greater in men [53,54].

Given resource limitations, the increased potential for male mice to fight and scratch, producing skin damage which would interfere with observations [55], meant that longer studies of skin edema, immune suppression, and tumour development after UV were only undertaken in female mice.

Topical NPS-2143, like 1,25D, produced a significant decrease in skin edema of mice, reflecting reduced inflammation after ssUV. UVR induces immediate and sustained production in NO in the skin [56,57,58,59] promoting the secretion of inflammatory mediators such as IL-6 [60]. A major limitation of the study is that it was not possible under the circumstances to examine a cytokine profile of mouse skin tissue before and after UV with or without NPS-2143 or 1,25D. Nevertheless, from the literature, there is evidence that NPS-2143 reduces NLRP3 inflammasome activation [61,62,63], overproduction of NO [64,65], the pro-inflammatory cytokine IL-6, and more [66,67,68,69]. These observations could explain the ability of NPS-2143 to reduce inflammation in mouse skin on day four after UV.

Somewhat surprisingly, despite a reduction in skin inflammation after UV, treatment with NPS-2143 had no effect on UV-induced immune suppression. Both DNA damage and increased IL-6 are important promoters of UV-dependent immune suppression [70]. Yet NPS-2143, like 1,25D, reduced DNA damage and, from the literature, also reduces IL-6 [66,67,68,69]. UV can directly damage antigen-presenting cells and promote the production of immunosuppressive cytokines such as IL-10 and IL-4 [71,72,73]. IL-10 is an anti-inflammatory cytokine and a potent immunosuppressant [74]. Secreted by UV-irradiated keratinocytes [75,76] and regulatory T cells [77], IL-10 not only prevents T cell expansion and activation but can also suppress other antigen-presenting cells [74]. It has been shown that reductions in CPD using liposomes containing T4N5 endonuclease led to reduced UV-immune suppression due to decreases in UV-upregulated IL-10 and TNF-α at both the mRNA and protein levels [78]. Furthermore, IL-10^−/−^ mice were protected against photocarcinogenesis [79]. Though not tested in this study, IL-10 was increased with NPS-2143 treatment in rats [66,69]. This may explain why NPS-2143 failed to prevent UV-induced immunosuppression, though it protected against CPD and inflammation.

Skin tumour development depends on a combination of DNA damage, inflammation, and immunosuppression [49,80]. CPD are a major contributor to UV-induced mutations, so reduced CPDs might lead to fewer UV-induced mutations [80] and thus fewer tumours. Furthermore, enhanced repair of CPDs has been shown to reduce skin cancer incidence in mice [81] and humans [82] and we previously found that NPS-2143 increased DNA repair in keratinocytes [26]. Based on these findings, it seemed possible that NPS-2143 would have some protective capacity at an early stage of photocarcinogenesis due to its ability to reduce DNA damage and inflammatory reactions in vivo. In the photocarcinogenesis study, however, a single concentration of NPS-2143 (2280 pmol/cm^2^) was not superior to vehicle, either in time to develop the first tumour (including benign papilloma) or in the total number of tumours per mouse. Although NPS-2143 significantly reduced SCC incidence at 24 weeks, the effect did not persist.

Although the failure of NPS-2143 to prevent UV-induced immune suppression may explain its failure to prevent tumours in the chronic UV study, other factors may be involved. Cyclic AMP Response Element Binding protein (CREB) is a transcription factor essential for basic cellular function and homeostasis [83]. CREB is activated by phosphorylation at Ser133 by various kinases [83,84]. CREB overexpression supports growth and progression in various cancers [85,86,87,88,89]. CREB activation promotes enhanced cell proliferation, dysregulation of differentiation and reduced sensitivity to apoptosis and metastasis, particularly in melanoma [87,90] and SCC [88,91]. Using human keratinocytes, we observed that UV exposure increased phosphorylation of CREB at Ser 133 (phospho-CREB Ser^133^). While it would have been useful to verify the CREB and p-CREB changes in mouse skin, this was beyond the scope of the study and is a limitation. Our results in human keratinocytes are consistent with a recent study using reverse phase protein microarray analysis, which reported that p-CREB Ser^133^ was significantly activated at 1 h, 5 h, and 24 h after a single acute dose of 2MED UV in human skin [92] and with the report of increased p-CREB in mouse skin [46].

It has been argued that p-CREB is important in the initiation of papilloma formation, while other transcription factors such as CCAAT/enhancer binding protein (C/EBP)–B [93,94] control later stages of tumour growth and Activator Protein 1 (AP1) [95,96,97] maintains tumour identity. In a study of SCC, shRNA-mediated knockdown of CREB resulted in a significant increase in G2 phase arrest and a reduction in tumorigenic activity [91]. These authors identified that a key transcription factor complex, CREB and RFX1, which binds in the nucleus and is stabilized by CCAR2, is required to maintain proliferation in SCC [91]. Overexpression of CREB in a human squamous carcinoma cell line SCC13 remarkably increased its colony forming ability via a β-catenin-dependent pathway [88]. These studies suggest critical functions of CREB not only in the initial stage of papilloma formation but also in the development of neoplastic characteristics of SCC. Treatment of keratinocytes with 1,25D reduced UV-induced expression of p-CREB, fitting with its ability to protect mouse skin from developing both papillomas and SCC in the photocarcinogenesis study. NPS-2143, on the other hand, did not reduce UV-upregulated p-CREB. This may be part of the explanation for its inability to reduce tumour incidence, apart from its failure to decrease UV-induced immunosuppression.

Bikle et.al reported that double knockout of the vitamin D receptor and CaSR in the epidermis leads to spontaneous SCC formation in mice without any induction by UV, which was not observed in mice with deletion of either gene alone [98,99]. Those studies did not involve UV exposure. This is the first study to investigate whether negative modulation of the CaSR in skin alters responses to UV. NPS-2143 reduced two types of DNA damage in epidermal cells as well as skin inflammation to a similar extent as 1,25D, a known photo-protective agent (Figure 1, Figure 2 and Figure 3). However, NPS-2143 did not ameliorate UV-induced immune suppression (Figure 3). This latter observation, together with the failure of NPS-2143 to reduce post-UV CREB phosphorylation, probably explain the limited effect of this compound on skin tumour formation after ssUV (Figure 4). It is possible that the reduction in UV-induced DNA damage including oxidative damage by NPS-2143 may indicate an anti-aging effect [100]. These novel findings may lead to new research directions on the relationship between UV and the CaSR.

## 4. Materials and Methods

### 4.1. Studies in Mice

**Figure 5:**
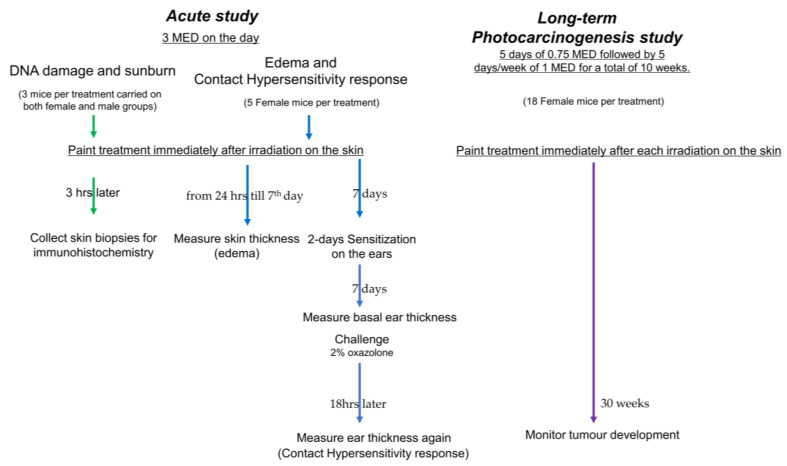
Experimental design scheme.

The in vivo studies were approved by the Animal Ethics Committee of the University of Sydney (Approval number: 2015/794) and conformed to ARRIVE criteria. Skh:hr1 hairless albino mice, originally from Charles River (Wilmington, MA, USA), were from an in-house colony maintained at the University of Sydney. All Skh:hr1 hairless mice were housed in groups in wire-topped plastic boxes at an ambient temperature of 23–25°C under gold lighting (F40GO tubes, General Electric Co., Hobart, TAS, Australia) that does not emit UV radiation, and fed with Gordon Rat and Mouse Pellets (Yanderra, NSW, Australia) and tap water ad libitum. Male and female Skh:hr1 mice that were aged-matched in groups were used for experiments [31]. Mice were not allowed to be housed singly for this study but were housed in groups. Female mice are less prone to fighting than male mice and the fighting produces skin damage and artefacts [55]. For this reason, it is possible to study both female and male mice for DNA damage within hours after a UV exposure; however, the use of female mice for studies of skin edema or contact hypersensitivity conducted over 7 days and 16 days, respectively, or photocarcinogenesis (over 40 weeks) is preferred (Figure 5).

As previously established, the minimum erythemal dose (MED) of UV with this source for Skh:hr1 mice was 1.33 kJ/m^2^ UVB and 23.7 kJ/m^2^ UVA [31,101]. UV-irradiated mice were subjected to a single dose of 3 MED of UV (UVB value at 3.99 kJ/m^2^) for acute and immunosuppression studies. In the chronic photocarcinogenesis study, mice were subjected to 5 days of 0.75 MED followed by 5 days/week of 1 MED, for a total of 10 weeks (Figure 5).

### 4.2. Topical Treatments, DNA Damage and Sunburn Cells

Mice were treated topically over approximately 7 cm^2^ on the irradiated dorsal skin with 100 μL of vehicle only, or vehicle containing 1,25D (Sapphire Bioscience Pty Ltd., Redfern, NSW, Australia), or NPS-2143 2143 (HY-1007 MCE^®^, Medchem Express, Monmouth Junction, NJ, USA) immediately after irradiation, as previously described [31]. The compounds (1,25D and NPS-2143) were freshly diluted in spectroscopic grade ethanol (Merck, Darmstadt, Germany), combined with propylene glycol (Sigma-Aldrich, St. Louis, MO, USA) and MilliQ water at a ratio of 2:1:1 (*v*/*v*/*v*). Vehicle (base lotion) was combined, ethanol:propylene glycol:water 2:1:1 *v*/*v* [31]. The dose of NPS-2143, equivalent to 20× (228 pmol/cm^2^) and 200× (2280 pmol/cm^2^) of an effective dose of 1,25D 11.4 pmol/cm^2^ was determined according to the same ratio of 1,25D doses as determined from in vitro experiments [26,31]. Biopsies of dorsal skin were taken in triplicate from each mouse, 3 h post-UV and paraffin-embedded for immunohistochemistry of DNA damage as previously described [31]. Quantification of positive nuclei as % total nuclei (the percentage of CPD or 8-OHd positive nuclei staining in the selected nuclei in an area of epidermis) was obtained using MetaMorph (Molecular Devices, San Jose, CA, USA) and normalized to SHAM.

Routine haematoxylin and eosin staining was carried out by Veterinary Pathology Diagnostic Service (University of Sydney) to visualize sunburn cells. The stained sections were examined under a Zeiss Axioscan light microscope (Oberkochen, Germany) at 20× magnification, and the number of sunburn cells per linear millimetre of skin section recorded, as previously described [31,47]. Non-irradiated samples as SHAM control were obtained from the abdomen. Three areas of each section were analysed.

### 4.3. Skin Edema and Induction of Contact Hypersensitivity in Mice

Changes in dorsal skin thickness, a measure of edema, were recorded daily from 24 h onward until the until levels returned close to pre-UV condition on the 7th day after irradiation.

The contact hypersensitivity response was tested to investigate the effects of NPS-2143 on UV-induced systemic immunosuppression, as previously described [31]. Briefly, female mice were sensitized 1 week after irradiation and treatments, with 100 μL of 2% oxazolone (Sigma-Aldrich, USA) (*w*/*v*) in absolute alcohol applied to the non-irradiated abdominal skin. Sensitization was repeated on the subsequent day. The sensitized mice were challenged 2 weeks after irradiation by application of 5 μL 2% oxazolone to both surfaces of each ear, so that each mouse received 20 μL in total. Ear thickness measurements, taken using a spring micrometre (Interapid, Zurich, Switzerland), were recorded before the challenge and at 18 h after challenge, as previously reported [31]. The difference between pre- and post-oxazolone challenge ear thickness measurements of each mouse was recorded as ear swelling and the means for each group of 5 mice was calculated. Ear Swelling = pre-challenge ear thickness–post-challenge ear thickness.

The immune response was then calculated for each mouse, as shown in the formula below:

(1) $$\mathrm{Immune}\;\mathrm{Response}\;=\frac{\mathrm{Ear}\;\mathrm{swelling}\;\mathrm{of}\;\mathrm{UV}\;\mathrm{IRRADIATED}\;\mathrm{mice}\;}{\mathrm{Ear}\;\mathrm{swelling}\;\mathrm{of}\;\mathrm{UV}\;\mathrm{NON}-\mathrm{IRRADIATED}\;\left(\mathrm{SHAM}\right)\;\mathrm{mice}}$$

Immunosuppression was calculated as 100% minus this value, ± SEM [31] as in the formula below:

Immunosuppression % = (1 − Immune Response) × 100(2)

### 4.4. Photocarcinogenesis

For this study, groups of 18 mice were used. Immediately after ssUV irradiation, mice were treated topically with either base lotion [31], 1,25D (11.4 pmol/cm^2^), or NPS-2143 (2280 pmol/cm^2^). During the next 30 weeks, the time of appearance, location, and visual identification of tumours with a diameter of at least 1 mm were monitored and mapped for each mouse. As previously described [31], the term “tumour” includes papilloma and SCC. The photocarcinogenic outcomes were reported as tumour latency, tumour incidence, tumour multiplicity, and SCC incidence. At the end of the experiment, all tumours were harvested for histological examination to confirm the classification.

### 4.5. Culture of Primary Human Keratinocytes

Keratinocytes were harvested from skin samples under University of Sydney Human Research Ethics Committee protocol no. 2015/063 and cultured, as previously described [26]. The concentration of NPS-2143 used in these in vitro studies was based on previous experiments where we performed serial dilutions of NPS-2143 to determine the concentration-dependent response in human keratinocytes [26].A total of 500 nM was in the effective concentration range (5 nM~500 nM) and was also 10× IC 50; thus, it was chosen for all the in vitro experiments.

### 4.6. Western Blot

Keratinocytes were irradiated with an Oriel 1000 W xenon arc lamp (Newport Corporation, USA) and subsequently treated with vehicle, 1,25D, or NPS-2143, as in our previous study [26]. Western blot was performed, as previously described [102], with α-tubulin as the loading control. Primary antibodies used in this study were anti-phospho-CREB (Ser133) at 1 in 1000 dilution (mouse monoclonal, #9196, Cell Signaling Technology, Trask Lane Danvers, MA, USA), anti-CREB(Total) at 1 in 1000 dilution (mouse monoclonal, #9197, Cell Signaling Technology, Trask Lane Danvers, MA, USA), or anti-tubulin at 1µg/mL (mouse monoclonal, SC-5286, Santa Cruz Biotechnology). The band was imaged with the ChemiDoc^TM^ imaging system (Bio-Rad Laboratories, Inc, Hercules, CA, USA) and densitometry was carried out using Image J. SHAM, showing negligible expression of p-CREB which served as a negative control, and the data was normalized to UV+ vehicle to pool experiments.

### 4.7. Statistical Analysis

Animals in this study were divided into treatment groups of three for acute study, groups of five for immunosuppression study, and groups of eighteen for chronic photocarcinogenesis [31,47,101,103]. These numbers were determined by power analysis manually calculated from data from previous studies to have an 80% chance showing a 20% difference between treatment group at a significance level of 5% [104,105]. Results are expressed as either as mean + SEM or as indicated. All the data sets passed goodness of fit tests, which determine whether sample data exhibit skewness and kurtosis that matches a normal distribution. All statistical analyses were performed with GraphPad Prism statistical program 9.0 (GraphPad Software Inc.). Unless otherwise stated, analysis of comparisons between treatment groups were made by linear mixed-model analysis, appropriate for dealing with repeated measurement data. The Mantel–Haenszel log-rank test (also called Mantel–Cox test) was used to analyse incidence data in the photocarcinogenesis study [31,45].

## Figures and Tables

**Table 1:** (a) Tumour Incidence and (b) SCC incidence analysed using a Mantel–Cox test.

(a) Total Incidence by Mantel-Haenszel Log Rank Analysis	Significance by Week 27	Significance by Week 28
**Vehicle vs. 1,25D 11.4 pmol/cm^2^**	**** (*p* < 0.0001)	**** (*p* < 0.0001)
**Vehicle vs. NPS-2143 2280 pmol/cm^2^**	* (*p* = 0.044)	n.s (*p* = 0.21)
**(b) SCC incidence by Mantel-Haenszel Log Rank Analysis**	**Significance by week 24**	**Significance by week 25**
**Vehicle vs. 1,25D 11.4 pmol/cm^2^**	**** (*p* < 0.0001)	**** (*p* < 0.0001)
**Vehicle vs. NPS-2143 2280 pmol/cm^2^**	* (*p* = 0.02)	n.s. (*p* = 0.15)

**Table 2:** A schematic representation of the differences between the effects of 1,25D and NPS-2143.

	In Vitro Photoprotection (Yang et al., 2022 [26])	In Vivo Photoprotection against UV-Induced DNA Damage and Sunburn	Protection against UV-Induced Acute Skin Inflammation	Protection against UV-Induced Immunosuppression	Prevent Skin Carcinogenesis
**1,25D**	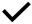	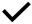	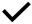	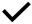	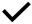
**NPS-2143**	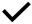	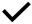	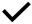	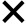	Delayed initiation but no long-term protection

## Data Availability

The data that support the findings of this study are available from the corresponding author upon reasonable request. Some data may not be made available because of privacy or ethical restrictions.

## Supplementary Materials

The following supporting information can be downloaded at: https://www.mdpi.com/article/10.3390/ijms24054921/s1.
