# Supplementary material for: The CaSR Modulator NPS-2143 Reduced UV-Induced DNA Damage in Skh:hr1 Hairless Mice but Minimally Inhibited Skin Tumours
Source: Int J Mol Sci. 2023 Mar 3;24(5):4921. doi: 10.3390/ijms24054921 (PMC10002576; doi:10.3390/ijms24054921)
Supplement: Supplementary file 1 [file ijms-24-04921-s001.zip › ijms-2132133-supplementary.pdf]

Supplementary Materials:

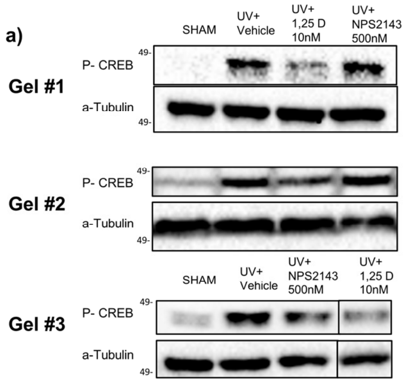

Densitometry	Gel #1	Gel #2	Gel #3		Gel #1	Gel #2	Gel #3	Mean	SD
SHAM	0.1	4959.3	2336.0		0.00	0.19	0.09	**0.09**	**0.09**
UV +Vehicle	66896.5	26491.8	26893.9	**Normalize to UV**	1.00	1.00	1.00	**1.00**	**0.00**
UV+D	51327.9	13878.8	15281.4		0.77	0.52	0.57	**0.62**	**0.13**
UV+NPS2143	72434.8	21761.1	31015.4		1.08	0.82	1.15	**1.02**	**0.17**

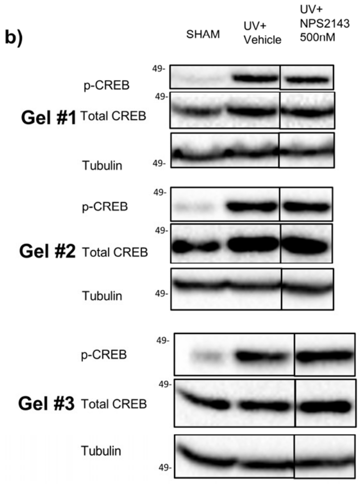

Gel #1	p-CREB	Total CREB	Tubulin	p-CREB/ T-CREB	Normalize to UV+Vehicle	p-CREB/ Tubulin	Normalize to UV+Vehicle
SHAM	2430.69	1922.75	3616.41	1.26	0.40	0.67	0.30
UV+Vehicle	8773.60	2805.75	3891.65	3.13	1.00	2.25	1.00
UV+NPS2143	5855.96	2323.53	3510.87	2.52	0.81	1.67	0.74
**Gel #2**							
SHAM	1408.23	12410.69	2992.28	0.11	0.46	0.47	0.46
UV+Vehicle	3172.04	12860.57	3110.16	0.25	1.00	1.02	1.00
UV+NPS2143	2818.63	12648.10	2929.16	0.22	0.90	0.96	0.94
**Gel #3**							
SHAM	775.04	1935.82	2485.06	0.40	0.48	0.31	0.29
UV+Vehicle	3025.16	3589.70	2851.63	0.84	1.00	1.06	1.00
UV+NPS2143	3014.46	2762.00	2405.04	1.09	1.30	1.25	1.18

Figure S1. Western Blot and densitometry of **a)** p-CREB and Tubulin.**b**) p-CREB, total CREB and tubulin. Human keratinocytes cultured in 96 well plates were irradiated with 400 mJ/cm^2 UVB followed by treatment with vehicle, 10nM 1,25(OH)$_2$D$_3$ or 500 nM NPS-2143 in the presence of 1 mM CaCl$_2$ for 90min. In pannel a) and b) blot was cut at the balck solid line to remove a lane tested on compound X (not to be disclosed). Triplicates blot and densitometry data in the table from b) were used to generate data in Figure 4f. Gel #1in b) is the repsentative blot for Figure 4f.

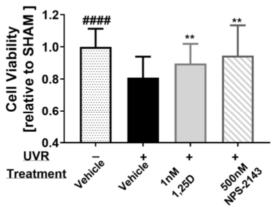

Figure S2 **Cell Viability by CTB assay** analysed at 3 hours post UV with treatments as indicated on primary keratinocytes Media contained 1.0 mM Ca^{2+}_{o}. Data was presented as fluorescence values (Mean +SEM) normalized to SHAM. n=5 ** $p<0.01$, $F(1.546, 32.47) = 6.870$ compared with UV+Vehicle by linear mix model. ####, $p<0.0001$, significantly different from UV+Vehicle by T-test ($t=5.180$, $df=42$).

Methods for supplementary Figure S1a

Cell viability by Cell Titer Blue Assay

Human keratinocytes were irradiated with an Oriel 1000W xenon-arc lamp solar simulator (Newport Corporation) and then treated with vehicle, 1, 25D and NPS-2143 as previously [26]. Cell viability was determined using the Cell Titer-Blue® Cell Viability Assay (Promega, USA) according to the manufacturer's instructions.
